# Transdiagnostic similarities and distinctions in brain networks associated with autistic social impairments: a prospective cohort study

**DOI:** 10.1186/s13229-025-00686-w

**Published:** 2025-11-19

**Authors:** Jennifer L. Bruno, Julia R. Plank, Samantha Leder, Evelyn M.R. Lake, Emily S. Finn, Tamar Green

**Affiliations:** 1 Department of Psychiatry and Behavioral Sciences, Stanford University, 1520 Page Mill Road Palo Alto, Stanford, CA 94305 USA; 2 Department of Radiology and Biomedical Imaging, Yale University, New Haven, CT USA; 3 Department of Biomedical Engineering, Yale University, New Haven, CT USA; 4 Wu Tsai Institute, Yale University, New Haven, CT USA; 5 Department of Psychological and Brain Sciences, Dartmouth College, Hanover, NH USA; 6 Department of Pediatrics, Stanford University, Stanford, 94305 USA

**Keywords:** Autism, Noonan syndrome, Connectome-based predictive modeling, Functional brain networks, Genetics

## Abstract

**Background:**

Despite high rates of autism spectrum disorder (ASD), understanding of pathophysiology is limited. The RAS-mitogen-activated protein kinase (RAS-MAPK) pathway plays a crucial role in ASD and is altered in children with Noonan syndrome (NS). Children with NS offer a unique model to disentangle genetic and neurological underpinnings of ASD.

**Methods:**

This study aimed to examine functional brain network anatomy underlying social impairments in children with NS (*n* = 28, mean age = 8.24), and tested generalizability of models developed in a non-syndromic cohort enriched for ASD (Autism Brain Imaging Data Exchange (ABIDE), *n* = 352, mean age = 11.0). Connectome-based predictive modeling (CPM) was applied to fMRI data to predict the severity of autism symptoms, indexed by the Social Responsive Scale (SRS), in children with NS. Next, we tested if a model developed to predict autism symptoms in an autism-enriched sample of children without NS (ABIDE) could predict autism symptoms in children with NS.

**Results:**

Predicted SRS scores were significantly associated with observed SRS scores in NS (r_s_ = 0.43, *p* = 0.011). Application of the predictive model generated in the autism-enriched cohort (ABIDE) significantly predicted observed SRS scores in NS (r_s_ = 0.46, *p* = 0.018). Predictive brain networks in both NS and the non-syndromic cohorts included subcortical-cerebellar networks and visual processing networks.

**Limitations:**

The size of our NS cohort is small, given the rarity of NS. However, the significant cross-dataset comparison yielded in this study suggests that use of large publicly available datasets can be useful in contextualizing smaller and harder to collect datasets in rare genetic syndromes.

**Conclusions:**

The presence of shared brain networks suggests converging patterns of functional connectivity underlying autism symptoms across diagnoses. These findings point to potential overlap between non-syndromic autism and NS and highlight the value of human genetic models for studying ASD. Future work investigating RAS-MAPK pathway dysregulation may further elucidate its contribution to autism-related brain function.

**Supplementary Information:**

The online version contains supplementary material available at 10.1186/s13229-025-00686-w.

## Background

Autism Spectrum Disorder (ASD) is a complex neurodevelopmental disorder characterized by deficits in social communication and restricted and repetitive behaviors [1]. One in 36 children U.S. meet criteria for ASD diagnosis [2], and increasing prevalence has been observed worldwide [3]. Despite ongoing research efforts to identify the brain basis of ASD [4], clinical care for children with ASD is limited due to challenges with accurate diagnosis, early identification and prediction of outcomes. This is partly due to the heterogeneity of ASD [5] and to the limitations of neuroscience methods used to date.

Understanding the genetic underpinnings of ASD holds potential to resolve challenges with heterogeneity and make strides toward understanding underlying pathophysiology. ASD is highly heritable and, most often, the genetic risk is conferred by common variants each with a small effect that cumulatively increase one’s risk [6]. While the genes implicated in ASD risk are diverse, they cluster in six main functional pathways [7, 8], one of which is the RAS-mitogen-activated protein kinase (RAS-MAPK) pathway. Originally identified for its role in oncogenesis [9], the RAS-MAPK pathway is now recognized as a critical regulator of neurodevelopment, influencing neuronal proliferation and differentiation [10–12]. Dysregulation of the RAS-MAPK pathway has been linked to social impairments and autism-related behaviors [13–15]. Studying children with RASopathies, genetic disorders associated with alterations along the RAS-MAPK pathway [16], offers a genetically defined model to examine how molecular variations affect brain network phenotypes and social behavior. Although signalopathies (genetic variants affecting a signalling pathway like RAS-MAPK) currently explain only a small proportion of ASD cases, the probability of identifying a signalopathy in individuals with ASD is two to twenty times higher than in the general population, highlighting their potential as informative genetic models [13].

Noonan syndrome (NS) occurs in 1:2000 and is the most common RASopathy. NS is caused most often by a germline mutation in the *PTPN11* or *SOS1* gene (~60% of cases) which in turn upregulates the RAS-MAPK signaling cascade [17, 18]. Children with NS consistently exhibit social deficits [19–21], such as difficulty maintaining friendships as well as rigid and repetitive behaviors, sensory sensitivity, and restricted interests [18]. Twelve to thirty percent of children with NS meet diagnostic criteria for ASD [18, 22, 23]. Existing neuroimaging literature indicates overlap across structural [24–26] and functional [27–29] brain mechanisms in NS and non-syndromic (idiopathic) ASD. However, studies in genetic model syndromes such as NS have limited sample sizes, due to the rare occurrence of the syndromes, thus computational modeling approaches suffer from overfitting and results lack generalizability.

The present study seeks to improve the clinical translational utility of neuroscience research by combining a genetics-first approach, in which we examine individuals with NS as a model for ASD, with large scale data and computational modeling to improve generalizability. The overarching goal is to identify functional brain networks that underlie social impairments in children with and without NS. Identifying similarities and differences will provide clues as to how much of the underlying neural endophenotypes are similar across children with NS, children with non-syndromic ASD and in children who are typically developing (TD). Linking neural circuitry with underlying social impairments may help inform the development of personalized treatment approaches. Furthermore, the inclusion of individuals with neurogenetic syndromes such as NS in mechanistic research is important both to ensure their representation and to better understand how findings generalize across diverse populations with ASD.

We applied connectome-based predictive modeling (CPM), a robust, data-driven computational approach [30] to functional MRI data in order to assess associations between brain connectivity and social impairment, measured via the Social Responsiveness Scale (SRS) [31]. Our primary hypothesis was that CPM would reveal a significant association between functional connectivity and SRS scores in children with NS. Secondly, we tested the hypothesis that a model developed to predict social impairment in children with and without ASD from the general population would generalize to predict social impairment in children with NS. To accomplish this second goal, we included large-scale data from the Autism Brain Imaging Data Exchange (ABIDE-II). Importantly, both the NS and non-syndromic cohorts were enriched for autism symptoms. Successful cross-diagnostic prediction of social impairment in the NS cohort would suggest generalizability of brain-behavior associations and similar functional brain networks underlying social impairment due to RAS-MAPK upregulation and non-syndromic causes.

## Methods

### Participants

#### NS cohort

Participants with NS were recruited to a single site from across the United States and Canada via caretaker-led organizations, physician referrals, and online advertisements. Eligible participants included 39 children with NS aged 4–12 years (mean = 8.44, SD = 2.20, 25 females). Participants were required to present results from genetic testing showing the presence of *PTPN11* (*N* = 29) or *SOS1* (*N* = 10) mutations. Complete inclusion and exclusion criteria are in the Supplement. This research was conducted at the Stanford University School of Medicine. The Institutional Review Board (IRB) approved all study procedures. Legal guardians completed informed consent. Participants over the age of 7 years completed assent. Children with NS were representative of the United States as illustrated in Supplementary Figure 1.

#### Non-syndromic cohort (ABIDE)

Data were gathered from the Autism Brain Imaging Data Exchange II (ABIDE) a publicly available multisite dataset of clinical, demographic, and resting-state fMRI data collected in children with ASD and typically developing (TD) [4]. Participants in the ABIDE dataset were recruited from clinics and the community from multiple sites around the world (fcon_http://1000.projects.nitrc.org/indi/abide/). The ABIDE cohort is enriched for ASD and the present study includes data from 122 children with non-syndromic ASD and 230 TD children. Local IRBs approved all study procedures and informed consent procedures were followed.

### Behavioral assessment

The Social Responsiveness Scale (SRS) was used in both NS and ABIDE to estimate social impairments and their severity, i.e., a higher score indicates greater severity of social impairment [31, 32]. The SRS is highly correlated Raw scores were utilized to maximize individual variability and avoid differences in scoring between SRS versions. We used the SRS Total score: a score of autistic social impairment calculated by summing the five SRS subscales of social awareness, social cognition, social communication, social motivation, and restricted and repetitive behaviors. A diagnostic interview, the Kiddie Schedule for Affective Disorders and Schizophrenia [KSADS]) [33] was completed for 26/28 children the NS cohort. Full scale IQ was assessed in the NS cohort via the Wechsler Intelligence Scale for Children (WISC) [34]. In the ABIDE, intelligence was assessed via the WISC, the Kaufman Brief Intelligence Test [35] or the Differential Abilities Scale [36].

### Image acquisition and preprocessing for the NS cohort

Prior to the MRI scan, all participants completed behavioral training in a mock MRI scanner to minimize motion and sensitivity to the scanner environment [37]. Imaging data were acquired on a GE Healthcare Discovery 3.0 Tesla whole-body MR system using a standard 8-channel head coil (GE Medical Systems, Milwaukee, WI). Whole-brain resting-state fMRI data were acquired using a T2-weighted gradient-echo spiral sequence and high-order shimming with an acquisition time (TA) of 6 minutes 8 seconds. Participants were instructed to relax and remain still. High-resolution T1-weighted structural images were acquired using a magnetization-prepared rapid gradient-echo (MPRAGE) sequence.

Image preprocessing was conducted using *fMRIPrep* 1.3.0.post3 (RRID:SCR_016216). To minimize the impact of motion artifacts, frames that were displaced by > 0.5 mm were eliminated along with the prior frame and the two subsequent frames [38]. To be included in analysis, imaging data was also required to meet the following two conditions: 1) the quality and orientation of structural and functional scans were deemed sufficient as outlined in registration procedures published by FMRIPrep https://fmriprep.org/en/1.0.3/api/index.html; and 2) a minimum of 4 minutes of functional data was artifact-free following frame elimination as described above. Following this procedure, twenty-eight of the 39 eligible participants were included in further analysis. Further imaging details are provided in the Supplement.

### Connectome-based predictive modeling (CPM) within each dataset

For each subject, a 268 × 268 connectivity matrix was calculated according to a previously defined functional atlas [39]. The connectivity matrix was calculated using Pearson correlation coefficients of the time-courses between node pairs, followed by application of Fisher’s r-to-z transformation. Each entry in the matrix represents an ‘edge’ i.e., the strength of the functional correlation between a node pair. The complete matrix represents the individual’s functional connectome. The matrices and SRS Total scores for each subject were entered into the connectome-based predictive modeling (CPM) procedure as described in detail in the Supplement [30]. Briefly, CPM is a data-driven predictive modeling approach that aims to identify patterns of functional brain connectivity that predict behavior (here, autistic social impairments quantified by the SRS Total score) in novel subjects. A leave-one-out cross-validation was used to build the predictive model by identifying edges significantly related to SRS Total scores while controlling for covariates of age and in-scanner motion. Positive and negative connectivity networks were constructed based on their association with SRS scores and a linear model was fitted to predict SRS scores for test subjects. Following all iterations of the leave-one-out cross-validation, model accuracy was computed by calculating the Spearman correlation between predicted and observed SRS scores. The accuracy of the model was evaluated using permutation testing (*n* = 1000 permutation) and *p*-values were generated based on the proportion of sampled permutations that were greater or equal to the true prediction corelation. Finally, functional brain networks were visualized using open-source BioImage Suite Web software (https://bioimagesuiteweb.github.io/webapp/) and BrainNet (https://www.nitrc.org/projects/bnv).

### Application of non-syndromic ABIDE models to the NS dataset

We leverage a predictive model that was previously generated for the non-syndromic cohort (ABIDE) [40] following the same steps as described for the NS cohort. The ABIDE model was built using combined data from participants with ASD (*n* = 122) and TD (*n* = 230). This model demonstrated a significant relationship between predicted and actual SRS Total scores (*r* = 0.32, *p* < 5E-10) for the population on which it was trained. Further details are described in the Supplement. The original CPM analysis of the ABIDE cohort and generation of the model is described in a previous paper [40]. Given that ABIDE is a multi-site dataset, a leave-one-site-out framework was tested and there was no significant change in findings. Furthermore, CPM predictions remained significant within the ASD group alone, indicating that the identified networks capture dimensional variation in symptom severity rather than merely reflecting categorical group differences.

### Network anatomy underlying autism symptoms

The network anatomy underlying SRS scores in the NS and ABIDE datasets was compared using the hypergeometric cumulative distribution function (hygecdf, MATLAB). The probability that *n* shared edges exist between the CPM-defined network and edges within or between 10 predefined functional atlas networks [39, 41] was calculated. To determine the extent to which the ABIDE and NS models share common features, the ABIDE and NS networks were compared at a ‘low threshold’ (any edge identified in any participant) and at a ‘high threshold’ (edges that appear in at least 90% of participants). The likelihood (1 – *p*-value) that each network (and between-network pair) contributed ABIDE and NS networks are reported in the Results.

## Results

Twenty-eight participants with NS were included following data scrubbing (mean framewise displacement after scrubbing = 0.147 mm, SD = 0.044 mm). We include ABIDE data from 122 ASD and 230 TD after data scrubbing [40]. Table 1 shows the demographics of the NS and ABIDE cohorts. As expected, the NS cohort demonstrated higher SRS Total scores than would be expected in a typically developing population, indicating more severe autism symptoms in NS (Supplementary Fig. 2). Two children had scores in the mild range indicating mild to moderate deficits in social interaction, 5 had scores in the moderate range indicating moderate to severe deficits in social interaction, and 5 has scores in the severe range, indicating clinically significant social behavior deficits that are strongly associated with ASD diagnosis. [42] The remaining 14 children had scores in the normal range. According to the KSADS, deficits in social-emotional reciprocity were observed in 4 children, while 3 demonstrated difficulties maintaining relationships. One child showed challenges in nonverbal communication.

**Table 1:** Descriptive statistics for children included in imaging analysis

Measure	All NS (n = 28)	ABIDE (n = 352)		
	Noonan (n = 28)	All (n = 352)	TD (n = 230)	ASD (n = 122)
Age (years)	8 (2), range 4–11	11 (2), range 6–15	10 (2)	11 (2)
Sex (male/female)	10/18	250/102	147/83	103/19
Full scale IQ (Standard score)	95.3 (12.9)	112 (15)	116 (12)	105 (17)
SRS Total raw score	55.1 (32.0)	43.7 (40.2)	18.6 (12.9)	91.2 (30.2)

Data presented as counts or mean (SD). Children in the NS group include *PTPN11* (*n* = 21) and SOS1 mutations (*n* = 7). All values are mean (SD) with the exception of sex which is N male/female. TD = typically developing. NS = Noonan syndrome. ABIDE = Autism Brain Imaging Data Exchange. ASD = autism spectrum disorder. Both datasets are enriched for autism symptoms. Subset of ABIDE dataset (*n* = 352) also reported in Lake et al. (2019)

In the restricted and repetitive behaviors domain, stereotyped speech or repetitive motor movements were noted in 2 children, and 2 exhibiting rigid adherence to routines and highly restricted interests. Increased or decreased reactivity to sensory input affected 7 children.

None of the children met criteria for the Social Communication domain, while three met criteria for the Repetitive Behavior domain. This highlights the heterogeneity of ASD-like symptoms within NS, with sensory and repetitive behavioral symptoms more prevalent than social communication symptoms in this sample.

### Prediction of SRS scores in NS

**Fig. 1:**
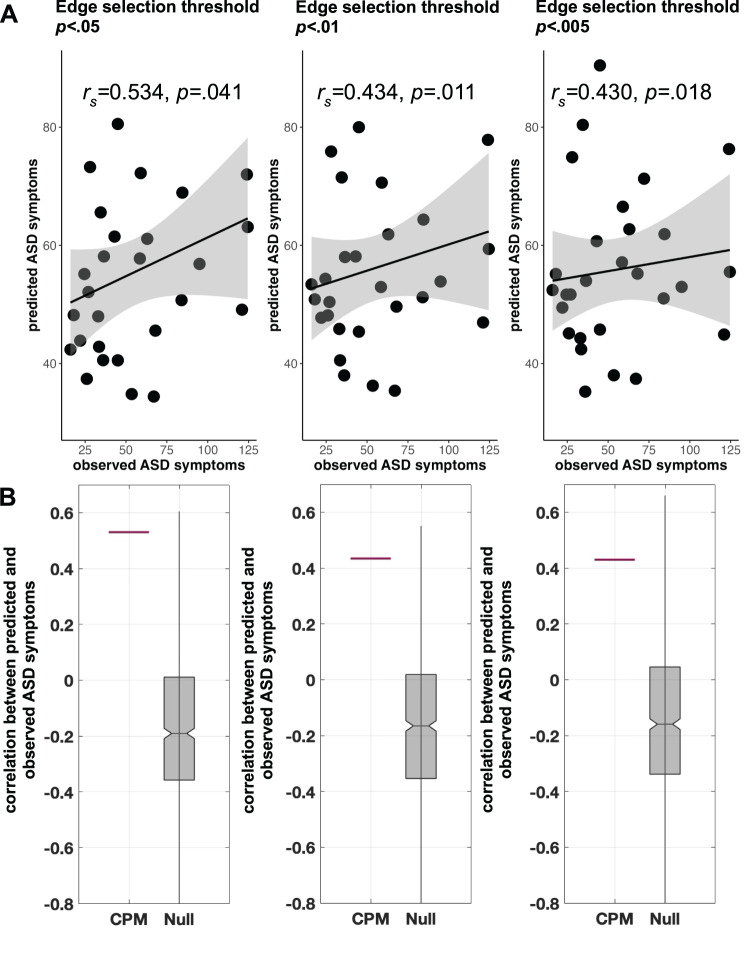
(**A**) Leave-one-subject-out cross-validation connectome-based predictive modeling (CPM) results in the noonan syndrome cohort. The sums of predicted SRS total scores from positive and negative models are plotted against observed SRS total scores at different initial edge selection thresholds of *p* < 0.05, *p* < 0.01, and *p* < 0.005. Spearman partial correlations (controlling for age and in-scanner motion) are presented; *p-*values indicate the significance from permutation testing (*n* = 1000). On each plot, black line is a linear regression line and gray indicates 95% confidence interval. (**B**) permutation testing indicated that correlations generated by CPM were higher than those generated by the null distribution across all edge thresholds. In each plot, the horizontal red line shows the correlation generated by leave-1-out cross-validation CPM. The grey box shows the correlations produced when subjects and SRS total scores are randomly shuffled prior to leave-one-out cross-validation, forming a null distribution (*n* = 1000 permutations). ASD = autism spectrum disorder; SRS = social responsiveness scale. Note that we used spearman correlation because we do not necessarily expect a linear relationship. Nonetheless, linear relationships are displayed here for visualization purposes

Predicted SRS scores were significantly associated with observed SRS scores at three edge selection thresholds: *p* < 0.05 (*r*_*s*_ = 0.53, *p* = 0.041; Fig. 1A), *p* < 0.01 (*r*_*s*_ = 0.43, *p* = 0.011), and *p* < 0.005 (*r*_*s*_ = 0.43, *p* = 0.018); *p* values were generated by comparing the correlations to the null distribution as described in the Supplement and thus serve as internal validation of the predictive model (Fig. 1B.) The predicted SRS scores at each edge threshold were similar to the actual SRS scores (*M* = 55.1, SD = 32.0) but had lower variance: *p* < 0.05 (*M* = 55.5, SD = 14.9), *p* < 0.01 (*M* = 56.2, SD = 14.2), and *p* < 0.005 (*M* = 55.9, SD = 13.5). The correlations and numbers of predictive edges at each threshold are provided in Supplementary Tables 2–4. Supplementary analysis, including FSIQ as a covariate (alongside age and motion), indicated similar results (Supplementary Tables 5–7).

To test specificity of the functional brain networks to social impairment, we tested whether the connectomes predictive of SRS Total scores could also predict FSIQ in NS (as described in the Supplement). The functional connectivity patterns did not significantly predict IQ in our NS cohort, suggesting the functional connections do not generalize across a broader cognitive domain and may be specific to social impairments.

### Functional connectivity profile of the predictive model in NS

**Fig. 2:**
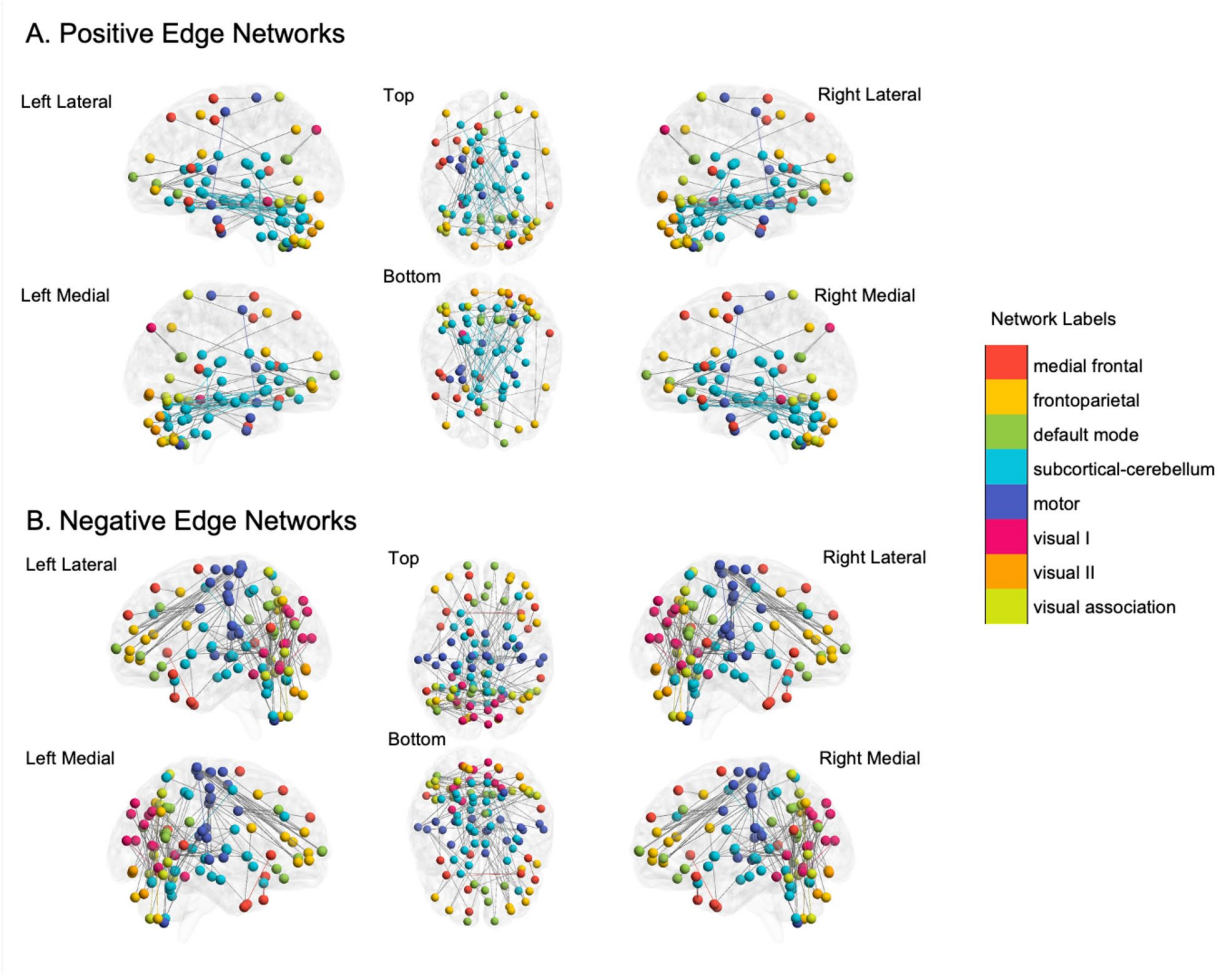
Functional networks predictive of SRS total scores in children with Noonan syndrome. Connectivity patterns for **A**. positive and **B**. negative edge networks generated by the predictive model are shown overlaid on multiple views of the brain. The network definitions are from Yeo, et al. [43]

To understand the functional distribution of the networks, the significant edges were divided into macroscale regions. The edge threshold of *p* < 0.01 was used for examination of the spatial distribution (Fig. 2, Supplementary Figure 3). The total number of significant positive, negative, and combined edges were 128, 214, and 342 edges, respectively. The greatest number of significant positive edges were between the subcortical-cerebellar networks (45 edges). The greatest number of significant negative edges were between visual area I-visual association area (47 edges), and the frontoparietal-motor areas (39 edges)

### Cross-dataset prediction: application of ABIDE model to NS

**Fig. 3:**
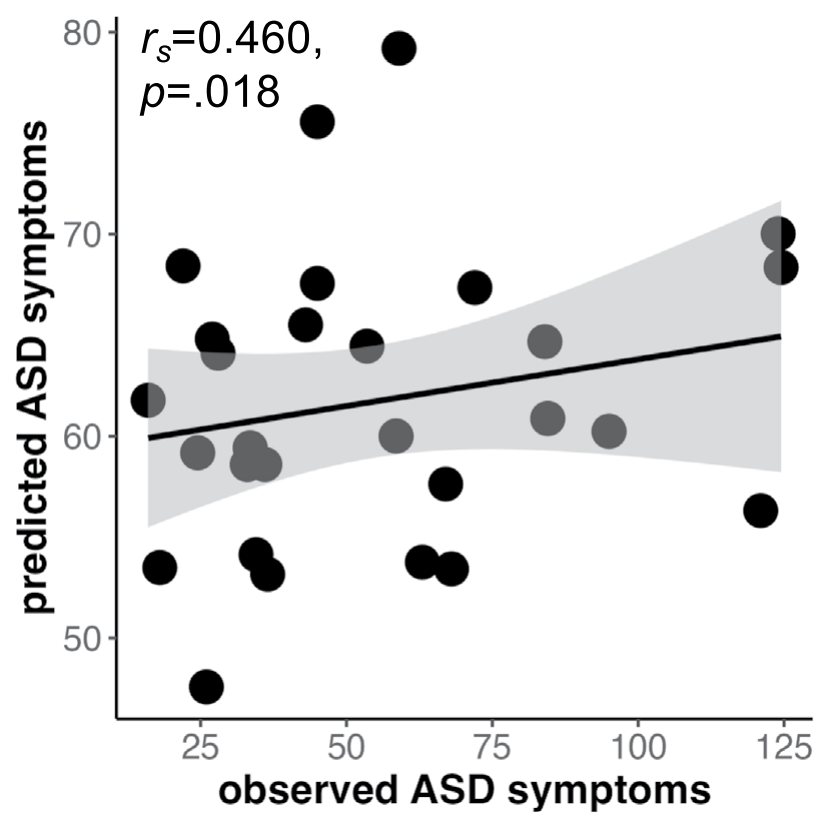
The predictive model generated using ABIDE predicts SRS total scores in an NS cohort. Spearman partial correlation including covariates of motion and age was used to assess the relationship between predicted and true SRS total scores in the NS cohort following application of the predictive model from ABIDE. We used spearman as a non-parametric correlation as we did not assume a linear relationship. Only edges significant in 90% of subjects were included in the model. ASD = autism spectrum disorder; NS = Noonan syndrome. ABIDE = autism brain imaging data exchange

The predictive model generated in ABIDE significantly predicted SRS scores in NS (*r*_*s*_ = 0.460, *p* = 0.018, Fig. 3). The relationship between predicted and observed SRS Total score in the NS cohort was significant at all tested edge selection thresholds (25%, 50%, 75%, 90%, 100%); Spearman partial correlations ranged from *r*_*s*_ = 0.438 to 0.460, *p* = 0.025 to 0.018 (Supplementary Table 8). Figure 3 shows the correlation at an edge selection threshold of 90%, meaning only edges significant in 90% of subjects were included. The functional networks predictive of SRS scores between NS and ABIDE at a 25% threshold are shown in Supplementary Figure 4.

### Consistency of model prediction between within NS models and ABIDE applied to NS models

We also compared the accuracy of model prediction when using within group NS CPM versus the ABIDE model to predict scores in the NS group. We looked at the deviation (difference) between the predicted SRS Total scores and the actual observed score (i.e., predicted score – observed score). When the SRS Total score predicted by NS CPM deviates from the actual score, the SRS Total score predicted by the ABIDE model shows a similar deviation from the actual score (*r*_*s*_ = 0.916, *p* < 0.001; Supplementary Figure 5).

### Anatomy of SRS networks in NS and ABIDE

**Fig. 4:**
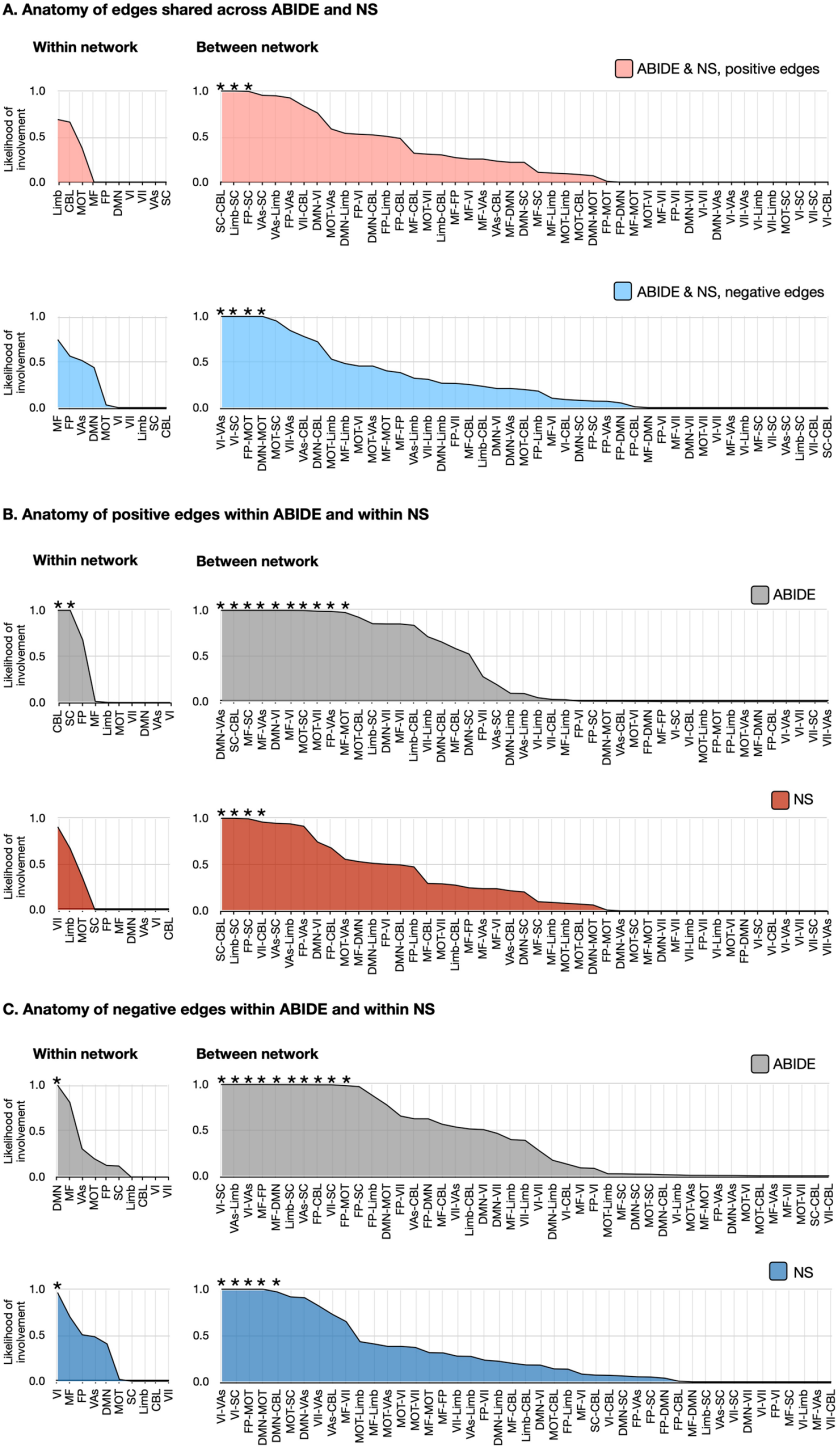
Anatomy of SRS networks in ABIDE (*n* = 352) and NS (*n* = 28). The shared edges appearing in both ABIDE and NS are summarized in (**A**) with positive edge networks in pink and negative edge networks below in blue. The anatomy of edges within ABIDE and within NS are summarized in (**B**) for positive edges, with ABIDE shown in gray and NS shown in red and in (**C**) for negative edges, with ABIDE shown in gray and NS shown in blue. These plots show the likelihood (1.0 – *p*-value) of edges being shared between a priori networks and the SRS network for 90% of participants in each of the cohorts. In all plots, networks and between-network pairs are ordered from greatest to least cumulative likelihood. These network representations were calculated using the hypergeometric distribution function in MATLAB. * indicates a likelihood greater than chance, i.e., *p* < 0.05. ABIDE, autism brain imaging Data Exchange; CBL, cerebellum; DMN, default mode network; FP, frontoparietal lobe; limb, limbic system; MF, mediofrontal cortex; MOT, motor areas; NS, Noonan syndrome; SC, subcortical areas; SRS, social responsiveness Scale; VAs, visual areas; VI, visual; VII, visual-II

Network edges were summarized according to a previously defined functional atlas [43] to capture the contribution of individual subnetworks. The likelihoods that each within-network or between-network edge contributed to social impairment networks are shown in Fig. 4 and Supplementary Figure 6. Shared and distinct network anatomy are described in Supplemental Results.

## Discussion

We probed the functional architecture underlying autism symptom severity in children with NS and a non-syndromic cohort, both enriched for ASD. Functional connectivity, particularly between subcortical-cerebellar and visual regions, significantly predicted autism symptom severity in children with NS. While our analysis was conducted on cohorts enriched for autism symptoms, the ability of the predictive model to significantly associate functional connectivity with social impairment severity across a spectrum, where some children demonstrate low or no social impairment while others exhibit severe social impairment, suggests connections may reflect general patterns of social impairment that transcend specific neurodevelopmental disorders. This emphasizes the relevance of such connectivity in understanding social behaviors more broadly. This neural circuitry may represent an endophenotype that links the effects of RAS-MAPK pathway pathogenic variants to autism symptoms and builds upon genetic studies which previously identified a role of the RAS-MAPK pathway in idiopathic ASD [8, 44]. Cross dataset prediction indicated significant overlap in the brain circuitry underlying autism symptoms in children with and without NS. Using models developed in the two independent cohorts, brain network similarities between NS and the general population were limited to between-network connections; within-network connections were not shared. These findings highlight the utility of examining relatively rare genetic syndromes in the context of large-scale data from the general population to disentangle the effects of specific pathways such as RAS-MAPK on brain functioning and resultant autism symptoms. This line of work holds promise for translating neuroscience findings into clinically meaningful breakthroughs.

### Examining the effects of RAS-MAPK pathway on functional connectivity and resulting autism symptoms

We demonstrate a unique pattern of neural circuitry underlying autism symptoms in children with NS. A significant proportion of the underlying network anatomy was localized to edges connecting subcortical and cerebellar regions, indicating a positive association between functional connectivity in these regions and social impairments. Subcortical regions are often highlighted in studies of NS due to structural and functional alterations. Structural MRI studies demonstrated reduced subcortical volumes in children with NS, specifically in the pallidum, putamen, caudate, thalamus, and hippocampus [45, 46]. Furthermore, advanced diffusion MRI models revealed reduced neurite density in these regions, suggesting impaired microstructural integrity, particularly in the hippocampus, putamen, and thalamus [47]. Complementing these structural findings, seed-based functional connectivity analyses indicate hypoconnectivity between subcortical regions in NS [29]. These results indicate convergence of structural and functional disruptions in subcortical regions in children with NS. Further research is needed to determine whether structural connectivity also contributes to social impairments, which could ultimately provide a more comprehensive understanding of the neural mechanisms underlying NS and ASD more broadly.

The network anatomy underlying autism symptoms in NS and ABIDE revealed weaker connectivity (negative edges) between primary visual and visual association areas, as well as between frontoparietal and motor areas, is associated with social impairments. Reduced connectivity in visual networks may contribute to deficits in processing and integrating visual information, aligning with known visuospatial impairments in NS [48]. Similarly, altered frontoparietal-motor connectivity may underlie motor coordination, executive function, and social difficulties. Given motor impairments in NS [49] and in ASD [50], future research may explore how these connectivity alterations evolve across development and link to visuospatial and motor coordination difficulties.

### Transdiagnostic links between neural circuitry and social impairment

Our results reveal a common positive predictive network of neuroanatomy, including subcortical and cerebellar regions, transdiagnostically in children with and without NS. This underscores the role of the cerebellum in the ‘social brain’ across a wide range of social impairment and suggests that cerebellar involvement may occur outside the involvement of the RAS-MAPK pathway. Accumulating evidence supports the involvement of the cerebellum in social functioning, and several studies have documented alterations in cerebellar structure among individuals with ASD [51–53]. In contrast, research exploring the cerebellum in NS has primarily concentrated on tumors, which are highly prevalent in NS [54, 55]. Our group’s diffusion MRI studies in NS identified significant structural alterations in the cerebellum, including reduced fractional anisotropy [26] and decreased neurite density relative to TD peers [47]. Children with ASD have demonstrated comparable reductions in fractional anisotropy [56, 57] and neurite density [58], indicating shared transdiagnostic structural alterations in the cerebellum. While this work was focused on functional connectivity, given these parallels, future research may explore the relationship between structural connectivity in NS and its implications for social impairment.

We also identified a common negative predictive network indicating that less connectivity was associated with greater social impairment severity. This network included primary visual and the visual association area, primary visual and the subcortical regions, frontoparietal and motor areas, and the default mode network (DMN) and motor area. Weaker connectivity has been found previously in children and adolescents with ASD, particularly in areas of the DMN [4]. Several studies have also found associations between weaker functional connectivity in the DMN and greater symptom severity [59, 60], (i.e., individuals with weaker DMN connectivity received higher scores on the Autism Diagnostic Observational Scale (ADOS) and the SRS-Total) [61]. However, a connectome-wide mega-analysis revealed networks of both hypo- and hyper-connectivity that correlated with social impairments in participants with ASD [62]. Taken together, these studies suggest hypo- and hyper-connectivity patterns are of importance for understanding social difficulties in ASD. Previous work in NS also revealed areas of both hyper- and hypo-connectivity [27, 29]; however, these studies did not investigate associations with autism symptoms. Our findings suggest similar patterns of predictive between-network connectivity in children with NS and ASD, suggesting non-syndromic samples may be leveraged to better understand the functional neuroanatomy underlying autism symptoms in NS.

A major distinction between NS and non-syndromic cohorts was the lack of convergence for within-network connectivity. Only between-network connectivity patterns were shared across cohorts. This finding speaks to the importance of examining connectivity patterns outside the boundaries of traditionally defined resting state networks, which is not always the norm in functional neuroimaging studies, including our recent work in NS [29]. In fact, within the NS cohort, the pattern of connectivity underlying autism symptoms primarily comprised between network connections. We previously found globally increased within-network connectivity for children with NS relative to children with TD and interpreted this as compensation for less efficient connectivity [29]. A predominance of between network and less within network connectivity, as found in the present study, may indicate additional reorganization due to inefficient network processing. Taken as a whole the divergence between connectivity patterns among NS and non-syndromic cohorts indicates the breadth of distinct neurobiological processes that may underlie autism symptoms in each condition.

## Limitations

Our findings identify functional neural circuitry that may represent an endophenotype linking the upstream effects of RAS-MAPK pathway pathogenic variants to downstream autism symptoms. However, we did not assay the RAS-MAPK pathway activity directly; future work could more directly link functional connectivity phenotypes to pathway activity through biochemical and genotype analyses. Image protocols and preprocessing were tailored to the unique aspects of each dataset and used rigorous data censoring, yet there were differences between the two cohorts. A recent preprint suggests resting-state fMRI pipeline variation, primarily regarding denoising, has limited effects on data outcomes [63] although we cannot rule out unintended effects. Furthermore, differences in templates and software choices may impact connectivity and study reproducibility [64]. While the original ABIDE CPM did not explicitly address harmonization across sites, we do note that the leave-one-site-out cross validation indicated site effects were not driving the results [40]. The age ranges of the NS (4 to 11 years) and non-syndromic (6 to 15 years) cohorts were different; however, age was included as a covariate in all analyses. Future work with more accurately matched age cohorts and longitudinal follow-up will be important to understand how networks underlying social impairments change with developmental processes. Although 10 out of 28 NS participants exhibited clinically significant autism symptoms based on the SRS or KSADS, none met full diagnostic criteria for ASD based on the KSADS. It is important to note that we did not include the ADOS (gold standard ASD assessment) and thus, results should be interpreted in the context of subthreshold ASD features within this cohort. Additionally, we did not compare functional connectivity patterns in children with NS to TD individuals. An examination of potential group differences could enhance our understanding of how functional connectivity in NS relates to that in TD individuals, and highlight the shared and distinct neural mechanisms underlying social behaviors. The NS cohort in this study included a higher proportion of females (*n* = 18, 64%) than males (*n* = 10, 36%) which may limit generalizability, particularly given the sex bias 4:1 towards males in the current ASD literature [65]. Future studies with larger sex-balanced cohorts will be important to examine potential sex-related effects on connectivity phenotypes. Finally, the size of our NS cohort is small, given the relative rarity of the NS mutations. Thus, we used a leave-one-out cross-validation and compared the result to a permutation-based null distribution. For a larger sample size, a split-half train-test split would be a more rigorous approach. However, the significant cross-dataset comparison yielded in this study suggests that use of large publicly available datasets (such as ABIDE) can be useful in contextualizing smaller and harder to collect datasets in rare genetic syndromes.

## Conclusion

Leveraging data-driven methodology, we uncovered similarities and distinctions in the predictive brain networks associated with greater social impairment severity among children with and without NS. The presence of shared brain networks suggests a converging pattern of functional connectivity underlying social deficits in both NS and non-syndromic ASD. According to recent genetic work, this pattern may signify the common involvement of the RAS-MAPK signaling pathway in ASD more broadly and points to treatments aimed at targeting RAS-MAPK as potentially promising therapeutic avenues.

## Electronic supplementary material

Below is the link to the electronic supplementary material.

Supplementary Material 1

## Data Availability

The final dataset collected for this manuscript will be stripped of identifiers and made available to qualified researchers. Data from the Autism Brain Imaging Data Exchange is available here: https://fcon_1000.projects.nitrc.org/indi/abide/.

## Abbreviations

ABIDE: Autism Brain Imaging Data Exchange
ASD: Autism spectrum disorder
CPM: Connectome-based predictive modeling
DMN: Default mode network
fMRI: functional magnetic resonance imaging
NS: Noonan syndrome
RAS-MAPK: Rat-sarcoma mitogen-activated protein kinase
SRS: Social Responsiveness Scale
TD: Typical developing
WISC: Wechsler Intelligence Scale for Children
